# Metabolomics Provides Insight into the Chemical Characteristics Underlying Bioactivity Differences Among Various Parts of *Atractylodes Chinensis* (DC.) Koidz.

**DOI:** 10.3390/ijms262211034

**Published:** 2025-11-14

**Authors:** Yehui Hu, Xiangui Mei, Yingying Cui, Zhenying Wang, Chuanzhi Kang, Shengci Fan

**Affiliations:** 1 Hebei Administration of Traditional Chinese Medicine Collaborative Innovation Key Research Laboratory of Chinese Medicinal Resource Industrialization, College of Traditional Chinese Medicine, Hebei University, Baoding 071000, China; 2 College of Agronomy, Shandong Agricultural University, Taian 271018, China; 3 State Key Laboratory for Quality Ensurance and Sustainable Use of Dao-di Herbs, National Resource Center for Chinese Materia Medica, Institute of Chinese Materia Medica, China Academy of Chinese Medical Sciences, Beijing 100700, China; kangchuanzhi1103@163.com

**Keywords:** *Atractylodes chinensis* (DC.) Koidz., metabolomics, different parts, chemical characteristics, bioactivity

## Abstract

*Atractylodes chinensis* (DC.) Koidz. (AK) is a kind of medicinal plant in the Asteraceae family, and its dried rhizomes have the functions of drying dampness, strengthening the spleen, dispelling wind and cold, and brightening the eyes. However, there remains insufficient development and utilization of other portions of the plant. To reveal the chemical characteristics and bioactivity potential of different AK parts, this study adopted UPLC-QE-MS/MS-based widely targeted metabolomics to analyze the metabolic components in ethanol extracts of AK rhizomes, fibrous roots, stems and leaves, flowers, and seeds. We then compared the antioxidant activities of these AK parts. The results showed that the highest ethanol extraction rate was from the rhizomes, while the flowers showed the strongest antioxidant activity. A total of 165 metabolites were categorized into seven major categories that included organic acids, flavonoids, and coumarins. Among these, organic acids were found with higher content in stems and leaves, fibrous roots, and seeds, while flavonoids were higher in flowers. This study explored the chemical composition and preliminary bioactivities of different AK parts based on widely targeted metabolomics. The results confirmed that the non-medicinal AK parts have high utilization values, and provided a scientific basis for the further development and utilization of this promising medicinal plant.

## 1. Introduction

*Atractylodes* is the dried rhizome of *Atractylodes lancea* (Thunb.) DC. or *Atractylodes chinensis* (DC.) Koidz. (AK) in the Asteraceae family. It has been widely used for centuries in China, South Korea, and Japan in the form of crude drugs [1]. *Atractylodes* plays an important role in both clinical and daily health care. It has the effect of drying dampness, strengthening the spleen, dispelling wind, dispersing cold, and brightening the eyes [2]. In addition to the clinical use of a large number of *Atractylodes* extracts, people often add *Atractylodes* to soups, porridges, and teas daily as part of a medicinal diet to improve their health. The market demand for *Atractylodes* has expanded in recent years, and the wild resources have been overexploited. Unfortunately, the plant’s natural recovery rate is much slower than the exploitation rate. In addition, the living environment of *Atractylodes* has been seriously damaged, and this has resulted in the imminent extinction of authentic *Atractylodes* resources. Moreover, a large number of cultivated varieties of *Atractylodes* have emerged. This has alleviated the shortage of its medicinal resources to some extent. However, there are significant differences in their medicinal bioactive ingredient efficacies due to differences in their origin and growth environments [3].

*Atractylodes* research has primarily focused on the chemical composition and pharmacological activity of the rhizome and its extracts, and the primary quality control indexes for *Atractylodes* are also focused on their volatile oils such as atractylodin, atractylon, and hinesol. Currently, little research has been conducted on the other parts of the plant. The traditional medicinal part of AK are the rhizomes, and during the harvesting process, its non-medicinal parts, such as the fibrous roots, stems and leaves, and flowers, are typically simply burned or buried as waste and are not fully utilized. This causes not only a great waste of resources, but also serious environmental pollution. Modern pharmacological studies have shown that the ethanol extract of *Atractylodes* has anti-inflammatory [4], anti-tumor [5], anti-gastric ulcer [6], digestion-promoting [7], and anti-pulmonary injury [8] effects. It has been shown that the extraction rate of AK stems and leaves could reach 27.81% by using 50% ethanol, and the ethanol extract was verified to promote the growth of tilapia and contribute to its intestinal health through the glycolytic pathway [9]. In addition, the AK roots contain certain volatile oils that have antibacterial activity. In addition, the stems and leaves, and flowers also contain flavonoids, total phenols, and total proteins [10].

Based on this, the aboveground parts of AK are important due to the production of metabolites that contain chemical components and their pharmacological activities that have certain similarities with the rhizomes. Therefore, the comprehensive utilization of AK resources to explore the utilization value of its non-medicinal parts is very necessary.

## 2. Results and Discussion

### 2.1. Extract Yield Determination of the Different AK Parts

**Figure 1:**
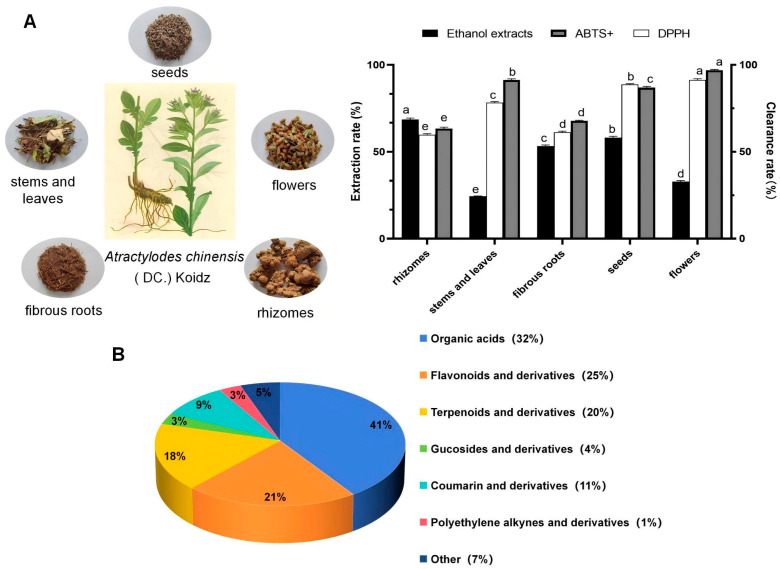
Different parts, extraction rates, and clearance rates of different parts in AK (**A**), metabolite classification (**B**). Different lowercase letters represent significant differences inextraction rate between different organs (*p* < 0.05).

The ethanol extract yields of the different AK parts were, in descending order, as follows: rhizomes > seeds > fibrous roots > flowers > stems and leaves. The highest ethanol extract yield of 68.572 ± 0.809% was in the rhizomes, followed by that in seeds with 58.124 ± 0.836%, and the lowest ethanol extract yield was 24.375 ± 0.206% in the stems and leaves (Table S1). Compared with the rhizome group, all other groups exhibited significant differences in ethanol extraction (Figure 1A).

### 2.2. Metabolomics Analysis of Different AK Parts

#### 2.2.1. Identification and Analysis of Chemical Components of Ethanol Extract from Different Parts in AK

In this study, based on the metabolomics technology of UPLC-QE-MS/MS, samples of five parts of R (rhizomes), FR (fibrous roots), SL (stems and leaves) and S (seeds), and F (flowers) from AK were examined and the composition of the substances was characterized, and 165 substances were screened and identified in positive and negative ion modes (Table S3). They were mainly categorized into 7 major types, including 67 types of organic acids, 35 types of flavonoids, 29 types of terpenoids, 15 types of coumarins, 5 types of polyacetylene components, 5 types of glycosides and 9 types of other components (Figure 1B).

#### 2.2.2. Principal Component Analysis (PCA), Heat Map, and Orthogonal Partial Least Squares Discriminant Analysis (OPLS-DA) of the Ethanol Extract from Different AK Parts

**Figure 2:**
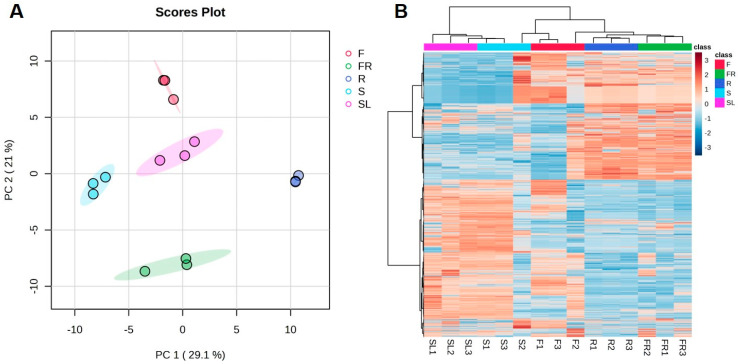
PCA score plots (**A**) and heat map (**B**) of 165 metabolites in different parts of AK (R, rhizomes; FR, fibrous roots; SL, stems and leaves; S, seeds; F, flowers).

Samples were analyzed by PCA in order to gain a preliminary understanding of the overall metabolite differences between the groups of samples and the magnitude of variability between samples within groups. Figure 2A shows that the contribution value of the first principal component (PC1) and the second principal component (PC2) was more than 50%, of which PC1 contributed 29.1% to the difference and PC2 contributed 21% to the difference characteristics, and the PCA results revealed that the AK flowers and seeds were clearly separated from the rest of the parts. These results suggested that the differential metabolites differed markedly between their sample groups. The AK stems and leaves, and the fibrous roots and rhizomes were more concentrated, suggesting that their differential metabolites might be more similar. In addition, the cluster heat map of all the detected substances in the five AK parts (Figure 2B) showed that the clustering was consistent among the three biological replicates of the different AK parts. Furthermore, the metabolic profiles were somewhat different among the different parts, demonstrating that the different AK parts had good reproducibility and credibility.

**Figure 3:**
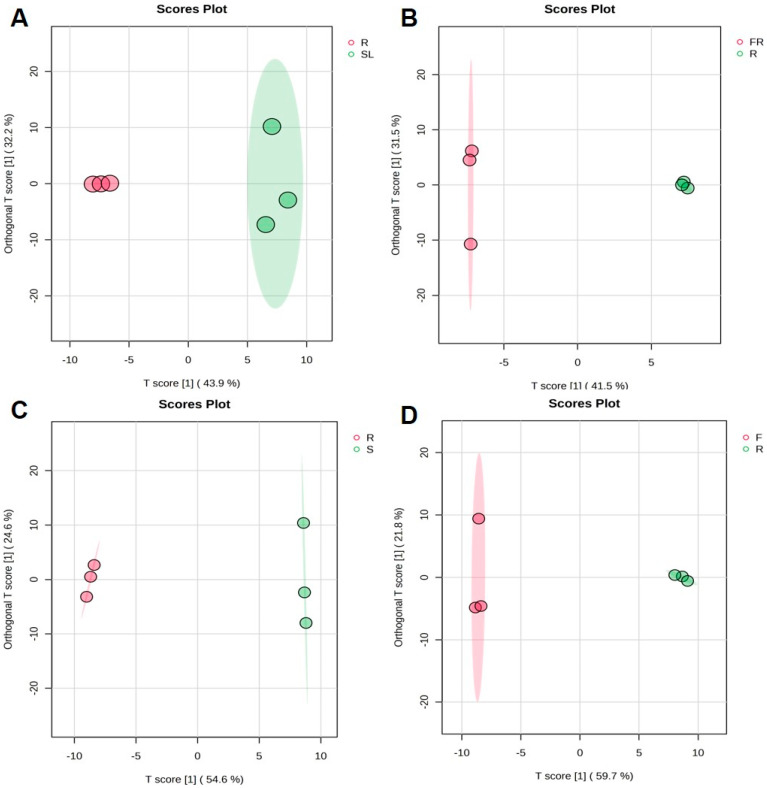
OPLS-DA score plots of different parts in AK: SL vs. R (**A**), FR vs. R (**B**), S vs. R (**C**), F vs. R (**D**) (R, rhizomes; FR, fibrous roots; SL, stems and leaves; S, seeds; F, flowers).

OPLS-DA is an extension of PLS-DA, and it effectively reduces model complexity metabolomics assay and enhances model interpretation without reducing the predictive power of the model to maximize the representation of between-group differences. To further screen the differential metabolic constituents among the different AK parts, comparative analyses of FR, SL, S, F, and R were performed to investigate the effects of different parts on the accumulation of metabolic constituents in AK. The results of the experiment were analyzed using OPLS-DA, and all the results proved that the model is suitable (SL vs. R: Q^2^ = 0.664, R^2^X = 0.429, R^2^Y = 0.794; FR vs. R: Q^2^ = 0.641, R^2^X = 0.378, R^2^Y = 0.789; S vs. R: Q^2^ = 0.918, R^2^X = 0.578, R^2^Y = 0.986; F vs. R: Q^2^ = 0.879, R^2^X = 0.0.597, R^2^Y = 0.940). The OPLS-DA score plot (Figure 3) showed that the sample points of different parts were obviously separated, while the points of the same type of samples were relatively concentrated, which indicated that there were some differences between the different ethanol extract samples, and the reproducibility between the same samples was good, indicating that the different parts of the samples had a significant effect on the metabolic constituents of AK ethanol extracts.

#### 2.2.3. Analysis of the Differential Metabolic Compositions: AK Stems and Leaves Relative to Rhizomes

**Figure 4:**
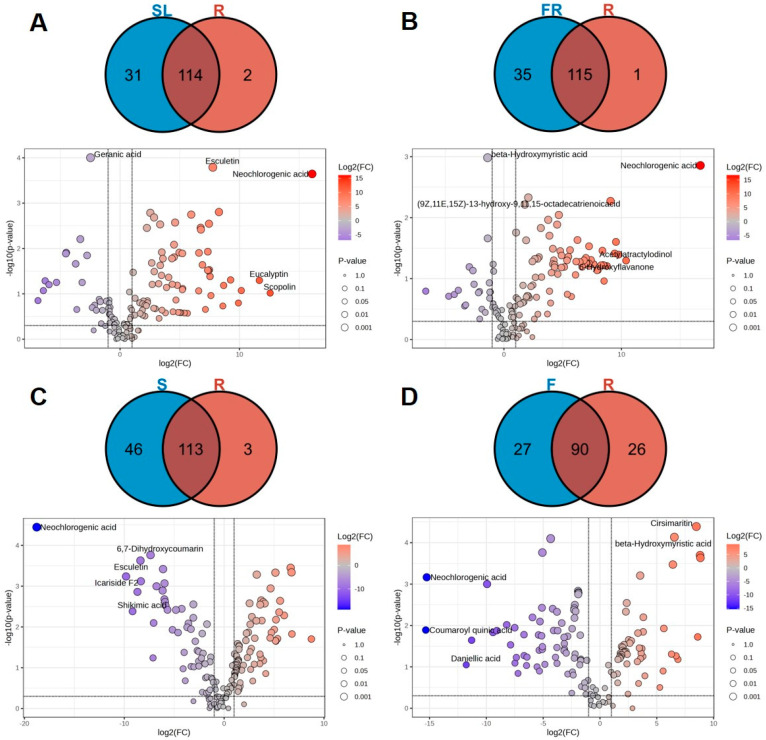
Volcano maps and Venn diagrams of different parts in AK: SL vs. R (**A**), FR vs. R (**B**), S vs. R (**C**), F vs. R (**D**).

The metabolites with top 20 differential multiplicities were screened, and a total of 40 metabolites were found. Of these metabolites, 34 were up-regulated and 6 were down-regulated for differential metabolic components (Figure 4A). The differentially expressed metabolites up-regulated in stems and leaves included 16 organic acids (cynarine, 1,3-dicaffeoylquinic acid, coumaroyl quinic acid, etc.); 5 flavonoids (Skullcapflavone II, quercetin, 7,4-dimethoxy-5-hydroxyflavanone, etc.); 4 types of terpenoids (farnesol, curdione, nardosinone, etc.); 3 coumarins (fraxin, aesculin, esculetin, etc.); 1 glucoside (icariside F2); 5 other components (paeonol, sinapoyl aldehyde, coniferyl aldehyde, etc.). Six differential metabolites were expressed downward in the stem and leaves. These included three organic acids (5-caffeoylquinic acid, myristic acid, N-methylthreonine, etc.); one polyacetylene (atractylodin); one flavonoid ((2Z)-6-hydroxy-2-[(4-hydroxy-3-methoxyphenyl)methylidene]-1-benzofuran-3-one); and 1 terpenoid (geranic acid).

The Venn diagram analysis (Figure 4A) showed that there were 114 differentiated metabolic components in the stems and leaves compared to the rhizomes, of which 10 differed in content and were dominated by organic acids. There were 31 unique differential metabolites in the stems and leaves, mainly dominated by flavonoids, of which there were 14 (wogonin, skullcapflavone II, 3-hydroxyflavone, etc.). It has been demonstrated that the flavonoids found in AK leaves are distinct from those found in AK flowers, with flavanols being the predominant type [10]. In addition to flavonoids, there were seven organic acids (cynarine, coumaroyl quinic acid, isochlorogenic acid A, etc.); three coumarins (aesculin, Fraxin, and 5,7-dihydroxycoumarin); and three terpenoids (daniellic acid, cyclodeca [b]furan-2 (3H)-one, and bakuchiol), of which the relatively high content is dominated by organic acids. There were two characteristic differential metabolites in the rhizomes, atractylodin and celastrol.

Organic acids are widely found in the roots, stems, leaves, and fruits of most herbs in plants, with antibacterial and anticoagulant effects, and they promote digestion and improve the immune function of the body, which is widely used in feed [11]. For example, the chlorogenic acid component of organic acids can restore colonic health by altering the structure of the intestinal microbial community [12] and inhibiting pro-inflammatory signaling [13]. In the present study, 67 organic acids were identified in different parts of AK. Some organic acid compounds (e.g., sinapic acid, shikimic acid, ferulic acid, chlorogenic acid, etc.) had decreased accumulation in the rhizomes and were enriched in the stems and leaves, seeds, and fibrous roots, and the contents were higher in the stems and leaves.

#### 2.2.4. Analysis of Differential Metabolic Compositions: Fibrous Roots Relative to Rhizomes in AK

The top 20 metabolites with multiplicative differences between the fibrous roots compared with the rhizomes were screened, and a total of 30 species were screened (Figure 4B). Among these, the components with greater differences in content were mainly flavonoids, and their contents were higher in the fibrous roots. Twenty-nine of them were up-regulated, and one was down-regulated. Thirty differential metabolites were upwardly expressed in the fibrous roots, of which fourteen were organic acid components (neochlorogenic acid, caffeoyl quinic acid, coumaroyl quinic acid, etc.); seven were flavonoids (puerarin, 3-hydroxyflavone, tangeritin, etc.); three were coumarins (6,8-dihydroxy-3-methyl-1H-2-benzopyran-1-one, esculetin and scopoletin); two were terpenoids (farnesol, curdione); and three were other components (5-(hydroxymethyl)-2-furancarboxaldehyde, sinapoyl aldehyde and coniferyl aldehyde et al.). One differential metabolite expressed downward in the fibrous roots was an organic acid (*β*-hydroxymyristic acid).

The results of the Venn diagram (Figure 4B) analysis showed that there were 115 differentiated metabolites in the fibrous roots and rhizomes. There were 35 different metabolites specific to the fibrous roots, including seven types—seventeen flavonoids (puerarin, 5-hydroxy-6,7-dimethoxyflavone, 3-hydroxyflavone, etc.); nine organic acids (caffeoyl quinic acid, coumaroyl quinic acid, quinic acid, etc.); two coumarins (aesculin and 5,7-dihydroxycoumarin); three glycosides (scopolin, icariside D1, and atractyloside A); two polyacetylenes (acetylatractylodinol and (6E, 12E)-tetradecadiene-8,10-diyne-1,3-diol-diacetate); one terpenoid (daniellic acid); and one other component (2-[(2E)-3, 7-dimethyl-2, 6-octadienyl]-4-methoxy-6-methylphenol). The characteristic differential metabolite in the rhizomes is one terpenoid component, celastrol.

The chemical profiles of the fibrous roots and rhizomes showed the highest similarity, with the difference being that the fibrous root-specific differential metabolites were dominated by flavonoid constituents and were relatively high in content.

#### 2.2.5. Analysis of Differential Metabolic Compositions: Seeds Relative to Rhizomes in AK

The top 20 metabolites with differential multiplicity were screened using the following conditions: *p* < 0.05, VIP > 1 and FC values ≥ 2 or ≤ 0.5. A total of 69 species were screened (Figure 4C), of which 32 were found to be up-regulated and 37 down-regulated. These were identified as differential metabolic components. A total of 32 differential metabolites were upwardly expressed in the seeds, of which 16 were organic acids (shikimic acid, sinapic acid, haematommic acid, etc.); 5 were terpenoids (farnesol, curdione, nardosinone, etc.); 3 were coumarins (fraxetin, 6,7-dihydroxycoumarin, and esculetin); 3 were flavonoids (gardenin A, hesperetin, and 5,6-Benzoflavone); 1 was glycoside (Icariside F2); and 4 were other components (paeonol, sinapoyl aldehyde, coniferyl aldehyde, etc.). A total of 37 differential metabolites were downwardly expressed in the seeds, including 11 organic acids (feruloyl quinic acid, myristic acid, valine, etc.); 11 terpenoids (thymol, myrcene, atractylenolide I, etc.); 6 flavonoids (quercetin cyanidin chloride, eucalyptin, etc.); 4 coumarins (aesculin, 6,8-dimethyl-4-hydroxycoumarin, umbelliferone, etc.); 3 polyacetylenes ((6E,12E)-tetradecadiene-8,10-diyne-1,3-diol-diacetate, diacetyl-atractylodiol, and atractylodin); and two other components (dibutylphthalate and cryptotanshinone).

The results of the Venn diagram (Figure 4C) analysis showed that there were 113 differential metabolites in seeds and rhizomes, of which the content varied considerably with organic acids dominating. There are 46 different metabolites unique to seeds, including 7 types, including 24 flavonoids (quercetin, cyanidin chloride, eucalyptin, etc.); 9 organic acids (feruloyl quinic acid, chlorogenic acid, 1,3-dicaffeoylquinic acid, etc.); 4 terpenoids (daniellic acid, bakuchiol, betulin, etc.); 4 glycosides (scopolin, syringing, icariside D1, etc.); 1 polyacetylene (acetylatractylodinol); and 1 other constituent (2-[(2E)-3,7-dimethyl-2,6-octadienyl]-4-methoxy-6-methylphenol). Among them, the relatively high content is flavonoid. There are three characteristic differential metabolites in the rhizomes, atractylodin, oleic acid, and 9-octadecenoic acid.

#### 2.2.6. Analysis of Differential Metabolic Compositions: Flowers Relative to Rhizomes in AK

To further screen for differential metabolic components in flowers compared with rhizomes, the screening conditions for differential metabolic components were set to *p* < 0.05, VIP > 1 and FC values ≥ 2 or ≤ 0.5. The metabolites with the top 20 multiplicity of differences were then screened and a total of 76 were identified (Figure 4D). Of these, 45 were up-regulated and 31 were down-regulated. A total of 45 differential metabolites were upwardly expressed in the flowers, including 20 organic acids (coumaroyl quinic acid, chlorogenic acid, shikimic acid, etc.); 12 flavonoids (5-hydroxy-6,7-dimethoxyflavone, sanggenone H, 3-O-methylquercetin, etc.); 9 coumarins (5,7-dihydroxycoumarin, 4-methylcoumarin, and 4-methylumbelliferone); 2 glycosides (scopolin and icariside F2); and 2 other constituents (sinapoyl aldehyde and coniferyl aldehyde). A total of 31 differential metabolites were downwardly expressed in the flowers, including 12 organic acids (2-hexadecenoic acid, valine, eicosenoic acid, etc.); 6 terpenoids (nardosinone, *d*-limonene, atractylenolide III, etc.); 6 flavonoids (4-methoxyflavone, 2-methoxyflavone, 5,7-dihydroxy-4-methoxyflavone, etc.); two polyacetylenes (atractylodin and (4E,6E,12E)-tetradecatriene-8,10-diyne-1,3-diol-diacetate); 1 coumarin constituent (7-hydroxy-4-methylcoumarin); and 2 other constituents (dibutylphthalate and paeonol).

The results of the Venn diagram (Figure 4D) analysis showed that there were 90 differentially metabolized components in the flowers and rhizomes, of which organic acids are predominant. There were 27 different metabolites unique to flowers, including 5 types, including 15 flavonoids (3-O-methylquercetin, 5,7-dihydroxy-4-methoxyflavone, quercetin, etc.); 6 organic acids (feruloyl quinic acid, nervonic acid, 1,3-dicaffeoylquinic acid, etc.); 3 coumarins (aesculin and 5,7-dihydroxycoumarin); 2 terpenoids (daniellic acid and bakuchiol); and 1 glycoside (scopolin), of which the flavonoid constituents were relatively high. There were 26 unique differential metabolites in the rhizomes, containing 6 types, including 8 terpenoids (selina-4(14),7(11)-dien-8-one, d-Limonene, atractylenolide III, etc.); 4 flavonoids (4-Methoxyflavone, 3,4-dimethoxy-7-hydroxyflavanone, 2-Methoxyflavone, etc.); 2 polyacetylene (atractylodin and (4E,6E,12E)-tetradecatriene-8,10-diyne-1,3-diol-diacetate); 2 coumarins (6,8-dihydroxy-3-methyl-1H-2-benzopyran-1-one and 7-hydroxy-4-methylcoumarin); and 2 other constituents (dibutylphthalate and paeonol).

Flowers are generally rich in flavonoids, and studies have shown that more flavonoids are identified in the flowers of AK than in its stems and leaves [14]. Additionally, it has been shown there is less similarity between the chemical constituents contained in the flowers of *Atractylodes* and the rhizomes. According to the literature, about 20% of plants contain flavonoids, especially in the flowers and leaves of plants. In pharmacology, it has anti-inflammatory, anti-cancer, anti-virus, anti-fatigue and anti-diabetic effects; in plant protection, it can enhance the role of plant resistance to protect the normal growth of plants under external stress; in the livestock industry, it is used as a feed additive to help improve the economic efficiency of livestock production [15]. The quercetin component of flavonoids has been shown to alleviate diabetic peripheral neuropathy [16], protect against cardiac damage [17], and attenuate neonatal hypoxic–ischemic brain damage [18].

#### 2.2.7. Enrichment Analysis of Differential Metabolic Components in Different Parts of AK

**Figure 5:**
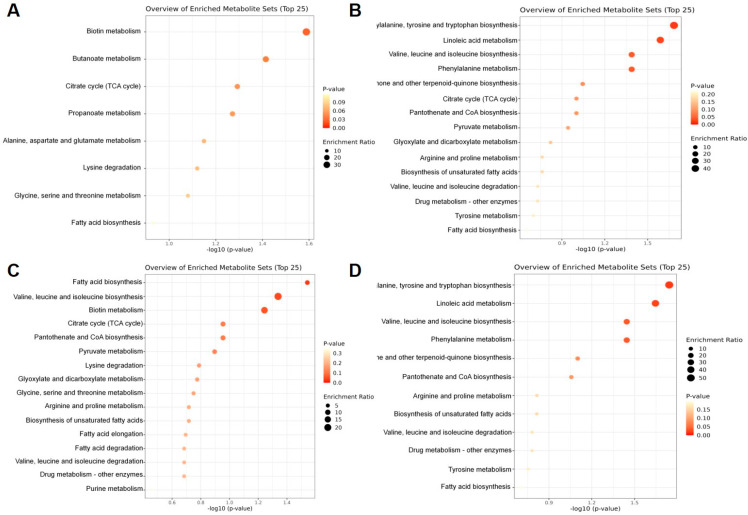
Enrichment bubble diagrams of metabolic pathways and antioxidant activity of differential metabolites in AK: SL vs. R (**A**), FR vs. R (**B**), S vs. R (**C**), F vs. R (**D**).

The number of differential metabolic components in the stems and leaves compared to the rhizomes (Figure 5A) was 40. Of these components, 36 were annotated using the Kyoto Encyclopedia of Genes and Genomes (KEGG) and imported into MetaboAnalyst, 8 metabolic pathways were annotated, and the top 5 pathways that contained the most differential metabolic components were biotin metabolism map00780, butanoate metabolism map00650, citrate cycle map00020, alanine, aspartate, and glutamate metabolism map00020, lysine degradation map00310. Biotin metabolism map00780 was the significantly enriched pathway (*p*-value < 0.05), followed by butanoate metabolism map00650 and succinic acid, and L-lysine components were up-regulated and expressed in stems and leaves.

The number of differential metabolic components in the fibrous roots compared to the rhizomes (Figure 5B) was 51. Of these, 46 were annotated using KEGG, and they were imported into MetaboAnalyst and annotated to four metabolic pathways, namely, biotin metabolism map00780, lysine degradation map00310, glycine, serine and threonine metabolism map00260, and arginine and proline metabolism map00330. Biotin metabolism map00780 was the significantly enriched pathway (*p*-value < 0.05), followed by lysine degradation map00310, with the lysine component being up-regulated and expressed in the fibrous roots, and the 4-hydroxyproline component in the rhizomes being up-regulated in the fibrous roots.

The AK seeds compared to rhizomes (Figure 5C) had 69 differential metabolic components. Of these, 56 had KEGG annotations, which were imported into MetaboAnalyst and annotated to 16 metabolic pathways. The top five pathways containing the most differential metabolic components were as follows: fatty acid biosynthesis map00061, valine, leucine and isoleucine biosynthesis map00290, Biotin metabolism map00780, Citrate cycle map00020 and pantothenate and CoA biosynthesis map00770. Fatty acid biosynthesis map00061 was the significantly enriched pathway (*p*-value < 0.05), followed by valine, leucine and isoleucine biosynthesis map00290. Palmitic acid, myristic acid, and valine components were up-regulated for expression in rhizomes.

The AK flowers were compared with rhizomes (Figure 5D), with 75 differential metabolic components, of which 62 were annotated with KEGG, and were imported into MetaboAnalyst, which annotated 15 metabolic pathways, with the top 5 pathways containing the most differential metabolic components being phenylalanine, tyrosine and tryptophan biosynthesis map00400, linoleic acid metabolism map00591, valine, leucine and isoleucine biosynthesis map00290, phenylalanine metabolism map00360, ubiquinone and other terpenoid–quinone biosynthesis map00130.

Phenylalanine, tyrosine and tryptophan biosynthesis map00400 was the significantly enriched pathway (*p*-value < 0.05), followed by linoleic acid metabolism map00591), with valine component up-regulated in flowers and tyrosine, 9,10-epoxy-12 (Z)-octadecenoic acid components up-regulated in rhizomes.

Flavonoids are a class of secondary plant metabolites widely found in herbs, fruits, and vegetables that cannot be synthesized directly by the human body and that must be ingested from external sources.

### 2.3. Determination of the Antioxidant Activities of Different AK Parts

It was shown that the antioxidant capacity of ethanol extracts from different parts of AK in the determination of the 2,2-diphenyl-1-picrylhydrazyl (DPPH) radical scavenging capacity were, in descending order, flowers > seeds > stems and leaves > fibrous roots > rhizomes (Table S2). The cation form of the compound 2,2′-azino-bis(3-ethylbenzothiazoline-6-sulfonic acid) (ABTS+) radical scavenging capacity assay showed that the antioxidant capacities of the ethanol extracts of different AK parts were in the following order, from high to low: flowers > stems and leaves > seeds > fibrous roots > rhizome (Figure 1A). The most significant component was the AK flower, which might be related to its flavonoid composition and content.

## 3. Materials and Methods

### 3.1. Materials and Instruments

The different parts of AK were collected from Hebei, China (117.46° E, 41.52° N) in October 2023 (Figure 1A). All the samples were deposited at Hebei University (Baoding, China).

Acetonitrile, methanol, and formic acid (HPLC grade) were purchased from Thermo Fisher Scientific (San Jose, CA, USA). Ammonium formate was obtained from Shanghai Yuanye Bio-Technology Co., Ltd. (Shanghai, China). The AK metabolites were analyzed using ultra-performance liquid chromatography–mass spectrometry (UPLC-QE-MS/MS).

### 3.2. Sample Preparation for Metabolomics

The samples obtained after the drying treatments were smashed, passed through a third sieve, and accurately weighed. The ultrasonic-assisted extraction was performed as follows: each sample was dissolved with 75% aqueous ethanol (20 mL per gram) and extracted at 30 °C with an ultrasonic bath (Scientz^R^ SB-5200DTD ultrasonic instrument, Ningbo, China). The ultrasonic power was 250 W, and the frequency was 40 kHz. After 40 min, the extracting solution was filtered using a 0.22 μm filter for analysis.

### 3.3. UPLC-QE-MS/MS Analysis Method

Analysis was performed with an Ultra-High-Performance Liquid Chromatography-Q Exactive Plus Orbitrap Mass Spectrometer (UPLC-QE-MS/MS) system (Thermo, Milford, MA, USA). The analysis procedures were performed with a Thermo Hypersil GOLD^TM^ AQ C18 column (150 × 4.6 mm, 5 μm) at constant temperature (30 °C). The mobile phases were selected as solvent A (0.01% formic acid in water and 2mM ammonium formate) and solvent B (acetonitrile and methanol in a 1:1 ratio). The elution gradient program was as follows: 0–1 min, 98%A; 1–25 min, 98–2%A; 25–27 min, 2%A; 27–27.1 min, 2–98%A; 27.1–35 min, 98%A. The injection volume was 10 μL and the flow rate was 0.30 mL/min.

The UPLC system was coupled with an Q Exactive Plus Orbitrap Mass Spectrometer (Thermo, Milford, MA, USA) equipped with an electrospray ionization source. Mass spectrometry was performed in both positive and negative ion modes using the following parameters: spray voltage, 3.5 kv; sheath gas, 40 arb; auxiliary gas, 10 arb; ion transfer tube temperature, 350 °C; resolution, 17,500; The powers of higher energy collisional dissociation are 20 eV, 40 eV and 60 eV. The mass spectra were viewed by Xcalibur^TM^ (Thermo, Milford, MA, USA).

### 3.4. Determination of Antioxidant Activity

Prepare a test solution with a concentration of 5 mg/mL for all sample groups and control groups.

DPPH powder weighed 7.13 mg, and 75% ethanol was added to 100 mL to form a DPPH solution at a concentration of 0.2 mmol/L. Sample group: take 1 mL of each extract sample of different parts of AK and DPPH reaction solution and mix well; Blank group: take 1 mL of each sample solution and 75% ethanol solution and mix well; Control group: take 1 mL of each DPPH solution and 75% ethanol solution and mix well. All treatments were shaken vigorously and mixed well, and then left to stand in a dark room for 30 min, and the absorbance was measured at the wavelength of 517 nm. ABTS powder 96 mg was weighed and added into purified water to 25 mL to formulate ABTS solution at a concentration of 7.4 mmol/L. The solution was prepared by adding purified water to 25 mL. K_2_S_2_O_8_ powder weighed 17.53 mg and was added into purified water to 25 mL to form a K_2_S_2_O_8_ solution with concentration of 2.6 mmol/L. The two were mixed well for 12 h, and then added with purified water to form K_2_S_2_O_8_ solution. PBS buffer was added until the absorbance at the wavelength of 734 nm was in the range of 0.7 ± 0.02, which was obtained as the working solution of ABTS +. Sample group: a total of 100 μL of extract sample of different parts of AK and 1 mL of the reaction solution of ABTS + were taken and mixed well; Blank group: a total of 100 μL of the sample solution was taken and mixed with 1 mL of purified water; Control group: a total of 1 mL of the solution of ABTS + was taken and mixed well. A total of 1 mL of ABTS + solution was taken and mixed with 100 uL of purified water. All treatments were mixed with 1 mL of water by shaking vigorously, and then left to stand in a dark room for 30 min, and the absorbance was measured at a wavelength of 734 nm. Vitamin C (VC) was used as a positive control. Calculate the clearance rate (%) using the following formula: Clearance rate (%) = [1 − (A_1_ − A_2_)/A_0_] × 100%, (A_0_: Blank group; A_1_: Sample group; A_2_: Control group).

### 3.5. Data Analysis

Chromatographic extraction was performed to analyze the major fragments using qualitative analysis software, and Thermo Xcalibur 2.1 software (Thermo Scientific, SanJose, CA, USA) was used to obtain the mass spectral information of all the compounds and to process the data. The extraction of the total ion flow diagram (TIC) was carried out using MZmine 2.53. The data were inverted using plethysmography, the peak alignment, and normalization using MS-DIAL to obtain the peak lists with data such as the m/z, retention time, and peak area, and the obtained data were matched against a database (MS/MS public vs17).

Principal component analysis (PCA) and orthogonal partial least squares discriminant analysis (OPLS-DA) were performed using MetaboAnalyst 5.0 (https://www.metaboanalyst.ca/ accessed on 1 January 2025) and Hip lot (https://hiplot.com.cn/ accessed on 1 January 2025). The screening conditions for the differential metabolites were as follows: *p* < 0.05, VIP > 1, and FC values ≥ 2 or ≤ 0.5, and the substances that met the conditions were considered to be differential metabolic components.

Pathway localization and enrichment of identified differential metabolic components were performed using the Encyclopedia of Genomes (KEGG) database (http://www.genome.jp/kegg/ accessed on 1 January 2025). All data were imported into SPSS 27. for univariate ANOVA analysis to assess their significance.

## 4. Conclusions

This study investigated the chemical characteristics and antioxidant activities of ethanol extracts from five parts of AK. The research showed that the rhizomes had the highest ethanol extraction rate, followed by flowers, seeds, fibrous roots, and stems and leaves. A total of 165 metabolites were identified through LC-MS based metabolomics, and these were classified into seven types that included organic acids, flavonoids, terpenes, and coumarins. There were significant differences in the chemical compositions from the different AK parts. Most metabolites were found at relatively high levels in the rhizomes and fibrous roots. Notably, flavonoids had the highest levels in the flowers and lower levels in the rhizomes. All five AK parts showed good antioxidant activities, among which the flowers had the strongest activity, which is most likely attributed to its high content of flavonoids. This study not only elucidated the differences in the metabolic compositions of the ethanol extracts from different tissue AK parts, and explored their specific expressions in the AK tissue parts, but also compared their antioxidants. The results of this study provide a reference for the comprehensive utilization and metabolic biosynthesis of AK resources.

## Data Availability

The original contributions presented in this study are included in the article/Supplementary Material. Further inquiries can be directed to the corresponding authors.

## Supplementary Materials

The following supporting information can be downloaded at https://www.mdpi.com/article/10.3390/ijms262211034/s1.
