# Supplementary material for: Metabolomics Provides Insight into the Chemical Characteristics Underlying Bioactivity Differences Among Various Parts of Atractylodes Chinensis (DC.) Koidz
Source: Int J Mol Sci. 2025 Nov 14;26(22):11034. doi: 10.3390/ijms262211034 (PMC12652469; doi:10.3390/ijms262211034)
Supplement: Supplementary file 1 [file ijms-26-11034-s001.zip › ijms-3948789-supplementary.pdf]

Supplementary materials

Metabolomics Provides Insight into the Chemical Characteristics Underlying Bioactivity Differences Among Various Parts from Atractylodes Chinensis (DC.) Koidz.

Yehui Hu [1], Xiangui Mei [2,3,*], Yingying Cui [2], Zhenying Wang [2], Chuanzhi Kang [3], Shengci Fan [1,*]

[1] Hebei Administration of Traditional Chinese Medicine Collaborative Innovation Key Research Laboratory of Chinese Medicinal Resource Industrialization /College of Traditional Chinese Medicine, Hebei University, Baoding 071000, China; 15395270255@163.com

[2] College of Agronomy, Shandong Agricultural University, Taian, 271018, China; 17860238022@163.com (Y.C.); 18766356851@163.com (Z.W.)

[3] State Key Laboratory for Quality Ensurance and Sustainable Use of Dao-di Herbs, National Resource Center for Chinese Materia Medica, Institute of Chinese Materia Medica, China Academy of Chinese Medical Sciences, Beijing, 100700, China; kangchuanzhi1103@163.com

* Correspondence: meixiangui@sdau.edu.cn (X.M.); fansc@hbu.edu.cn (S.F.)

Table. S1. Results of the extraction of chemical components from different parts of AK

NO.	Parts	Yield of ethanol extract%
1	rhizomes	68.572±0.809
2	stems and leaves	24.375±0.206
3	fibrous root	53.237±0.671
4	seeds	58.124±0.836
5	flowers	32.821±0.623

(Note: n=3, all other parts of AK reached significant levels of difference $p < 0.05$ when compared to the rhizomes.)

Table. S2. Results of antioxidant bioactivity of ethanolic extracts from different parts of AK

NO.	Parts	Clearance rate of DPPH %	Clearance rate of $ABTS^{+}$ %
1	rhizomes	$59.752±0.685^{f}$	$63.333±0.816^{f}$
2	stems and leaves	$78.296±0.612^{c}$	$91.358±0.617^{c}$
3	fibrous roots	$61.121±0.704^{e}$	$67.850±0.319^{e}$
4	seeds	$88.796±0.422^{b}$	$86.946±0.678^{d}$
5	flowers	$91.290±0.771^{a}$	$96.984±0.363^{a}$
6	VC	$75.901±0.453^{d}$	$94.595±0.351^{b}$

(Note: n=3, all other parts of AK reached significant levels of difference $p < 0.05$ when compared to the rhizomes.)

Table. S3. Results of UPLC-QE-MS/MS analysis of different parts of AK

Peak	t_R (min)	Molecular formula	Ion mode	Measured (m/z)	Theoretical (m/z)	ppm	Chemical compound
1	0.428	$C_4H_6O_5$	$[M-H]^-$	133.013	133.012	1.579	malic acid
2	0.652	$C_7H_6O_4$	$[M+Na]^+$	177.010	177.010	-0.678	protocatechuic acid
3	0.715	$C_{15}H_{20}O_2$	$[M+H]^+$	233.149	233.150	-2.788	atractylenolide ii
4	0.881	$C_{18}H_{32}O_2$	$[M-H]^-$	115.002	115.003	1.826	maleic acid
5	1.049	$C_5H_9NO_3$	$[M-H]^-$	130.049	130.050	-2.845	2-propanamidoacetic acid
6	1.052	$C_6H_{14}N_2O_2$	$[M+H]^+$	147.112	147.113	-5.914	l-lysine
7	1.059	$C_4H_6O_4$	$[M-H]^-$	117.018	117.019	-8.802	succinic acid
8	1.098	$C_7H_8N_4O_2$	$[M+Na]^+$	203.052	203.050	7.387	theobromine
9	1.119	$C_7H_{12}O_6$	$[M-H]^-$	191.055	191.056	-7.851	quinic acid
10	1.127	$C_5H_9NO_4$	$[M-H]^-$	146.044	146.045	-6.847	l-glutamic acid
11	1.168	$C_{10}H_8O_4$	$[M-H]^-$	190.977	190.977	3.456	6,8-dihydroxy-3-methyl-1h-2-benzopyran-1-one
12	1.231	$C_5H_9NO_2$	$[M+H]^+$	116.070	116.071	-1.809	l-proline
13	1.232	$C_6H_{11}NO_2$	$[M+H]^+$	130.086	130.086	0.307	pipecolate
14	1.306	$C_{19}H_{18}O_5$	$[M+H]^+$	327.115	327.116	-2.904	eucalyptin
15	1.326	$C_6H_{11}NO_2$	$[M+H]^+$	130.086	130.087	-8.840	dl-pipecolinic acid
16	1.332	$C_6H_5NO_2$	$[M+H]^+$	124.039	124.040	-7.820	isonicotinic acid
17	1.345	$C_{17}H_{14}O_7$	$[M+H]^+$	331.077	331.076	3.776	quercetin 3,7-dimethyl ether
18	1.351	$C_6H_6O_6$	$[M-H_2O-H]^-$	173.008	173.009	-7.976	aconitic acid
19	1.397	$C_6H_{13}NO_2$	$[M+H]^+$	132.139	132.140	-7.568	l-leucine
20	1.506	$C_8H_8O_2$	$[M-H]^-$	135.043	135.044	-4.073	phenylacetic acid
21	1.793	$C_6H_6O_3$	$[M+H]^+$	127.039	127.039	-2.991	5-(hydroxymethyl)-2-furancarboxaldehyde
22	1.998	$C_{15}H_{26}O$	$[M+H]^+$	261.094	261.094	-2.566	farnesol

23	2.140	$C_9H_{11}NO_3$	$[M+H]^+$	182.080	182.081	-4.229	tyrosine
24	2.942	$C_{10}H_{16}O_2$	$[M+NH_4]^+$	186.148	186.149	-4.781	chrysanthemic acid
25	3.070	$C_5H_5N_5$	$[M+H]^+$	136.061	136.062	-4.483	adenine
26	3.959	$C_9H_{11}NO_2$	$[M+H]^+$	166.086	166.086	-3.492	phenylalanine
27	4.028	$C_{20}H_{18}O_6$	$[M+Na]^+$	377.101	377.100	1.909	sanggenone h
28	4.427	$C_{10}H_{18}O$	$[M+H]^+$	155.142	155.143	-4.383	linalool
29	5.384	$C_{16}H_{18}O_9$	$[M-H]^-$	353.087	353.087	-0.255	chlorogenic acid
30	5.595	$C_8H_8O_4$	$[M-H]^-$	167.034	167.035	-8.381	vanillic acid
31	5.610	$C_6H_{11}NO_2$	$[M+H]^+$	130.086	130.086	-2.152	pipecolic acid
32	5.640	$C_{19}H_{12}O_2$	$[M+H]^+$	273.093	273.091	8.495	5,6-benzoflavone
33	6.103	$C_{11}H_{12}N_2O_2$	$[M+H]^+$	205.096	205.097	-5.071	tryptophan
34	6.328	$C_{16}H_{18}O_9$	$[M-H]^-$	353.087	353.088	-2.322	neochlorogenic acid
35	6.707	$C_{16}H_{12}O_7$	$[M+H]^+$	334.088	334.088	-0.928	3-methylquercetin
36	6.825	$C_{16}H_{18}O_{10}$	$[M+Na]^+$	393.078	393.080	-5.444	fraxin
37	6.893	$C_8H_8O_2$	$[M-H]^-$	135.044	135.045	-9.849	3-methylbenzoic acid
38	6.916	$C_{10}H_{18}O_4$	$[M+Na]^+$	225.109	225.110	-6.086	sebacic acid
39	7.121	$C_{13}H_{22}O_4$	$[M+Na]^+$	265.142	265.141	3.130	(z)-2-octylpent-2-enedioic acid
40	7.179	$C_9H_6O_4$	$[M+H]^+$	179.033	179.033	0.112	5,7-dihydroxycoumarin
41	7.280	$C_{15}H_{10}O_3$	$[M-H]^-$	237.057	237.057	0.000	3-hydroxyflavone
42	7.313	$C_{15}H_{16}O_9$	$[M-H]^-$	339.071	339.073	-3.775	aesculin
43	7.354	$C_{16}H_{18}O_9$	$[M+Na]^+$	377.083	377.080	6.869	scopolin
44	7.354	$C_{22}H_{42}O_2$	$[M-H]^-$	355.101	337.310	2.056	cryptochlorogenic acid
45	7.367	$C_7H_{10}O_5$	$[M-H]^-$	173.044	173.046	-8.437	shikimic acid
46	7.382	$C_9H_8O_4$	$[M+H]^+$	181.049	181.049	-4.087	caffeic acid
47	7.424	$C_9H_8O_5$	$[M-H]^-$	195.029	195.029	-2.307	haematommic acid
48	7.555	$C_{17}H_{20}O_9$	$[M-H]^-$	367.103	367.103	-2.070	feruloyl quinic acid

49	7.671	$C_{10}H_8O_4$	$[M+H]^+$	193.049	193.050	-6.527	scopoletin
50	7.773	$C_9H_{10}O_3$	$[M+H]^+$	167.069	167.070	-5.507	paeonol
51	7.873	$C_{16}H_{12}O_3$	$[M+H]^+$	253.106	253.105	1.936	4-methoxyflavone
52	7.904	$C_{20}H_{20}O_7$	$[M+Na]^+$	395.109	395.110	-3.088	tangeritin
53	8.156	$C_{18}H_{26}O_{10}$	$[M-H]^-$	401.145	401.147	-6.008	icariside f2
54	8.264	$C_9H_6O_4$	$[M-H]^-$	177.018	177.018	-1.695	6,7-dihydroxycoumarin
55	8.321	$C_9H_{10}O_5$	$[M-H]^-$	197.044	197.045	-4.111	syringic acid
56	8.371	$C_9H_8O_3$	$[M+H-H_2O]^+$	147.043	147.044	-6.121	coumaric acid
57	8.392	$C_9H_6O_4$	$[M-H]^-$	177.018	177.019	-7.739	esculetin
58	8.427	$C_{16}H_{18}O_8$	$[M-H]^-$	337.092	337.092	0.534	coumaroyl quinic acid
59	8.521	$C_{19}H_{28}O_{10}$	$[M-H]^-$	415.160	415.159	1.831	icariside d1
60	8.682	$C_{19}H_{18}O_8$	$[M-H]^-$	373.093	373.093	1.126	skullcapflavone ii
61	8.894	$C_{10}H_8O_5$	$[M+H]^+$	209.044	209.044	-4.592	fraxetin
62	9.040	$C_{22}H_{24}O_{10}$	$[M-H]^-$	285.077	285.077	-0.526	isosakuranin
63	9.253	$C_{16}H_{14}O_5$	$[M+NH_4]^+$	304.116	304.118	-5.721	(2r,3r)-3,7-dihydroxy-6-methoxy-2-phenyl-2,3-dihydrochromen-4-one
64	9.292	$C_{17}H_{16}O_5$	$[M-H]^-$	299.092	299.092	0.000	7,4-dimethoxy-5-hydroxyflavanone
65	9.381	$C_6H_{14}N_4O_2$	$[M+H]^+$	175.118	175.119	-5.596	arginine
66	9.502	$C_{21}H_{22}O_9$	$[M-H]^-$	313.069	313.071	-8.401	gardenin a
67	9.536	$C_{17}H_{14}O_5$	$[M+H]^+$	337.065	337.063	4.717	5-hydroxy-6,7-dimethoxyflavone
68	9.602	$C_{17}H_{14}O_5$	$[M-H]^-$	311.095	311.095	-0.418	3,4-dimethoxy-7-hydroxyflavanone
69	9.748	$C_{25}H_{24}O_{12}$	$[M+H]^+$	517.132	517.134	-4.119	1,3-dicaffeoylquinic acid
70	9.837	$C_{25}H_{24}O_{12}$	$[M+H]^+$	517.131	517.134	-5.859	cynarine
71	9.852	$C_6H_{10}O_5$	$[M-H]^-$	161.044	161.045	-7.079	hydroxymethylglutaric acid
72	9.872	$C_{11}H_{12}O_5$	$[M-H]^-$	223.060	223.061	-3.900	sinapic acid
73	9.916	$C_{10}H_{10}O_4$	$[M-H]^-$	193.049	193.050	-3.160	ferulic acid

74	10.038	$C_7H_6O_3$	$[M-H]^-$	137.023	137.024	-9.268	3-hydroxybenzoic acid
75	10.271	$C_{10}H_{16}O_2$	$[M+H]^+$	169.122	169.121	4.257	geranic acid
76	10.281	$C_{14}H_{28}O_2$	$[M-H]^-$	353.100	353.100	-0.620	5-caffeoylquinic acid
77	10.487	$C_{10}H_{10}O_3$	$[M+H]^+$	179.070	179.070	-2.736	coniferylaldehyde
78	10.497	$C_{11}H_{12}O_4$	$[M+H]^+$	209.080	209.081	-3.922	sinapoyl aldehyde
79	10.509	$C_{16}H_{14}O_6$	$[M+H]^+$	303.085	303.086	-2.409	5,7-dihydroxy-2-(4-hydroxy-3-methoxyphenyl)-2,3-dihydrochromen-4-one
80	10.652	$C_{16}H_{14}O_4$	$[M-H]^-$	269.082	269.082	0.111	8-hydroxy-5-methoxyflavanone
81	10.777	$C_{16}H_{12}O_7$	$[M+H]^+$	317.064	317.065	-2.965	3-o-methylquercetin
82	10.819	$C_{17}H_{24}O_9$	$[M-H]^-$	371.133	371.135	-4.365	syringing
83	10.837	$C_9H_{16}O_4$	$[M-H]^-$	187.096	187.097	-6.948	nonanedioic acid
84	10.92	$C_5H_6N_2O_2$	$[M-H]^-$	125.034	125.034	-3.279	4-imidazoleacetic acid
85	11.022	$C_{11}H_{10}O_3$	$[M-H]^-$	189.055	189.056	-7.247	6,8-dimethyl-4-hydroxycoumarin
86	11.123	$C_{18}H_{16}O_7$	$[M-H]^-$	343.081	343.082	-0.700	eupatilin
87	11.553	$C_{15}H_{22}O$	$[M+H]^+$	219.173	219.174	-4.106	selina-4(14),7(11)-dien-8-one
88	11.683	$C_{15}H_{22}O$	$[M+H]^+$	219.175	219.174	5.019	nootkatone
89	11.781	$C_{20}H_{24}O_4$	$[M+H]^+$	329.170	329.170	0.273	crocetin
90	11.836	$C_{17}H_{24}O_3$	$[M+H]^+$	277.178	277.180	-6.747	8β-ethoxyasterolid
91	11.892	$C_{16}H_{12}O_3$	$[M+H]^+$	331.042	331.041	3.867	2-methoxyflavone
92	11.901	$C_9H_6O_3$	$[M+H]^+$	163.038	163.038	-1.779	umbelliferone
93	11.960	$C_{16}H_{12}O_5$	$[M-H]^-$	283.060	283.061	-2.720	wogonin
94	12.040	$C_{16}H_{14}O_6$	$[M-H]^-$	301.071	301.072	-4.683	hesperetin
95	12.058	$C_{15}H_{10}O_7$	$[M-H]^-$	301.035	301.035	-2.824	quercetin
96	12.213	$C_{21}H_{36}O_{10}$	$[M-H]^-$	447.222	447.224	-5.188	atractyloside a
97	12.259	$C_{10}H_{16}$	$[M+H]^+$	137.132	137.132	-4.448	d-limonene
98	12.265	$C_{23}H_{24}O_{11}$	$[M+H]^+$	477.137	477.139	-3.835	cirsimarin

99	12.450	$C_{15}H_{22}O_3$	$[M+Na]^+$	273.141	273.140	2.673	nardosinone
100	12.549	$C_{17}H_{14}O_7$	$[M+H]^+$	331.080	331.082	-7.370	jaceosidin
101	12.815	$C_{11}H_{20}O_4$	$[M-H]^-$	215.128	215.129	-6.136	undecanedioic acid
102	12.985	$C_{18}H_{24}O$	$[M+H]^+$	257.188	257.190	-6.027	bakuchiol
103	13.158	$C_{10}H_{16}$	$[M+Na]^+$	159.110	159.110	0.440	myrcene
104	13.292	$C_{15}H_{24}O_2$	$[M+H]^+$	237.184	237.185	-4.722	eudesm-4(15),7-diene-9α,11-diol
105	13.529	$C_{30}H_{48}O_6$	$[M+Na]^+$	527.329	527.330	-2.788	polygalacic acid
106	14.061	$C_{21}H_{20}O_9$	$[M-H]^-$	415.102	415.103	-1.686	puerarin
107	14.071	$C_{10}H_8O_3$	$[M-H]^-$	175.040	175.040	0.343	7-hydroxy-4-methylcoumarin
108	14.075	$C_{16}H_{12}O_5$	$[M-H]^-$	285.076	285.076	-1.263	5,7-dihydroxy-4-methoxyflavone
109	14.081	$C_{18}H_{34}O_5$	$[M-H]^-$	329.233	329.233	-2.521	(z)-5,8,11-trihydroxyoctadec-9-enoic acid
110	14.085	$C_{15}H_{11}ClO_6$	$[M+H]^+$	323.030	323.030	-1.145	cyanidin chloride
111	14.140	$C_{16}H_{18}O_9$	$[M-H]^-$	353.087	353.08	-4.050	caffeoyl quinic acid
112	14.398	$C_{15}H_{24}O_2$	$[M+Na]^+$	259.161	259.160	2.624	curdione
113	14.409	$C_{17}H_{14}O_6$	$[M-H]^-$	313.071	313.072	-2.651	cirsimaritin
114	14.733	$C_{15}H_{20}O_3$	$[M-H]^-$	247.133	247.134	-4.451	atractylenolide iii
115	14.756	$C_{15}H_{18}O_2$	$[M+H]^+$	231.130	231.130	2.120	atractylenolide i
116	15.006	$C_{21}H_{22}O_8$	$[M+H]^+$	403.207	403.205	4.390	nobiletin
117	15.461	$C_{26}H_{32}O_5$	$[M+H]^+$	425.228	425.230	-4.868	licoricidin
118	15.629	$C_{15}H_{12}O_3$	$[M+H]^+$	241.085	241.086	-4.438	acetylatractylodinol
119	15.629	$C_{15}H_{12}O_3$	$[M+H]^+$	241.085	241.086	-4.438	6-hydroxyflavanone
120	15.820	$C_{10}H_{14}O$	$[M+H]^+$	151.111	151.112	-7.081	thymol
121	15.915	$C_{15}H_{26}O_3$	$[M-2H_2O+H]^+$	219.173	219.174	-2.509	5-hydroxyculmorin
122	16.077	$C_{13}H_{10}O$	[M+H]+	183.080	183.078	8.958	atractylodin
123	16.352	$C_{15}H_{24}$	$[M+H]^+$	205.160	205.160	-1.462	α-copaene
124	16.487	$C_{17}H_{20}O_4$	$[M-H]^-$	287.127	287.129	-7.767	diacetyl-atractylodiol

125	16.744	$C_{15}H_{20}O_2$	$[M-H]^-$	231.138	231.139	-4.673	cyclodeca[b]furan-2(3h)-one
126	17.170	$C_{16}H_{12}O_6$	$[M-H]^-$	299.055	299.055	0.100	5,7-dihydroxy-2-(4-hydroxyphenyl)-3-methoxy-4h-chromen-4-one
127	17.224	$C_{18}H_{22}O_4$	$[M+NH_4]^+$	320.184	320.186	-4.404	(6e,12e)-tetradecadiene-8,10-diyne-1,3-diol-diacetate
128	17.418	$C_{16}H_{22}O_4$	$[M+H]^+$	279.158	279.159	-4.943	dibutylphthalate
129	17.448	$C_{10}H_8O_3$	$[M-H]^-$	175.038	175.039	-4.342	4-methylumbelliferone
130	17.570	$C_{15}H_{22}O$	$[M+H]^+$	219.173	219.174	-5.201	α-cyperone
131	17.824	$C_{18}H_{30}O_3$	$[M-H]^-$	293.212	293.212	-2.694	(9z,11e,15z)-13-hydroxy-9,11,15-octadecatrienoicacid
132	18.230	$C_{20}H_{28}O_3$	$[M+Na]^+$	339.189	339.190	-2.418	daniellic acid
133	18.514	$C_{16}H_{12}O_5$	$[M-H]^-$	283.060	283.060	0.353	oroxylin a
134	18.528	$C_{14}H_{28}O_3$	$[M-H]^-$	243.196	243.196	-1.439	beta-hydroxymyristic acid
135	18.944	$C_{29}H_{38}O_4$	$[M-H]^-$	449.266	449.270	-7.323	celastrol
136	19.784	$C_{30}H_{50}O_3$	$[M+Na]^+$	481.362	481.360	4.113	soyasapogenol b
137	20.024	$C_{18}H_{32}O_3$	$[M-H]^-$	295.226	295.228	-4.979	9,10-epoxy-12(z)-octadecenoic acid
138	20.759	$C_{18}H_{20}O_4$	$[M+H]^+$	301.144	301.143	3.254	(4e,6e,12e)-tetradecatriene-8,10-diyne-1,3-diol-diacetate
139	20.823	$C_{10}H_8O_2$	$[M+H]^+$	161.059	161.060	-5.464	4-methylcoumarin
140	21.106	$C_{14}H_{28}O_2$	$[M-H]^-$	227.200	227.201	-1.276	myristic acid
141	21.264	$C_{20}H_{32}O_2$	$[M-H]^-$	303.232	303.232	1.286	arachidonic acid
142	21.310	$C_{18}H_{32}O_2$	$[M-H]^-$	279.232	279.233	-4.584	linoelaidic acid
143	21.386	$C_{16}H_{30}O_2$	$[M-H]^-$	253.216	253.217	-4.542	palmitoleic acid
144	21.656	$C_{16}H_{12}O_5$	$[M-H]^-$	283.060	283.060	0.000	(2z)-6-hydroxy-2-[(4-hydroxy-3-methoxyphenyl)methylidene]-1-benzofuran-3-one

145	21.853	$C_{25}H_{24}O_{12}$	$[M-H]^-$	515.118	515.120	-3.688	isochlorogenic acid a
146	21.894	$C_{30}H_{48}O_3$	$[M-H]^-$	455.352	455.352	-0.856	ursolic acid
147	22.333	$C_{17}H_{32}O_2$	$[M-H]^-$	267.232	267.233	-2.507	2-hexadecenoic acid
148	22.661	$C_{18}H_{34}O_2$	$[M-H]^-$	281.248	281.249	-3.164	oleic acid
149	22.672	$C_{18}H_{34}O_2$	$[M-H]^-$	281.249	281.249	1.422	9-octadecenoic acid
150	22.706	$C_{16}H_{32}O_2$	$[M-H]^-$	255.232	255.233	-5.799	palmitic acid
151	23.958	$C_{24}H_{48}O_2$	$[M-H]^-$	367.357	367.359	-6.642	lignoceric acid
152	24.117	$C_{17}H_{34}O_2$	$[M-H]^-$	269.248	269.250	-5.980	heptadecanoic acid
153	24.276	$C_{20}H_{38}O_2$	$[M-H]^-$	309.279	309.280	-3.945	eicosenoic acid
154	25.857	$C_{18}H_{26}O_2$	$[M+H]^+$	275.199	275.200	-3.198	2-[(2e)-3,7-dimethyl-2,6-octadienyl]-4-methoxy-6-methylphenol
155	25.950	$C_{10}H_{16}O$	$[M+H-H_2O]^-$	135.116	135.117	-7.179	carveol
156	26.496	$C_{30}H_{48}O_3$	$[M-H]^-$	455.353	455.353	-0.132	oleanoic acid
157	27.719	$C_{24}H_{46}O_2$	$[M-H]^-$	365.341	365.343	-3.996	nervonic acid
158	27.840	$C_{30}H_{50}O_2$	$[M-H]^-$	441.373	441.374	-2.764	betulin
159	28.383	$C_{22}H_{44}O_2$	$[M-H]^-$	339.326	339.326	0.000	behenic acid
160	28.610	$C_4H_6O_6$	$[M-H]^-$	149.008	149.009	-1.946	tartaric acid
161	28.786	$C_6H_6N_2O$	$[M+H]^+$	123.055	123.055	-3.169	niacinamide
162	29.041	$C_{12}H_{12}O_5$	$[M-H]^-$	234.982	234.981	2.724	5,6,7-trimethoxycoumarin
163	29.248	$C_5H_{11}NO_2$	$[M+H]^+$	118.086	118.086	-3.811	valine
164	29.648	$C_5H_9NO_3$	$[M+H]^+$	132.065	132.066	-4.165	4-hydroxyproline
165	29.690	$C_5H_{11}NO_3$	$[M+H]^+$	134.081	134.081	-3.953	n-methylthreonine
